# Comparative Performance Evaluation of Multi-Type LiDAR Sensors and Their Applicability to Sidewalk HD Mapping

**DOI:** 10.3390/s26051480

**Published:** 2026-02-26

**Authors:** Dongha Lee, Sungho Kang, Jaecheol Lee, Junghyun Kim

**Affiliations:** 1 Department of Civil Engineering, Kangwon National University, Chuncheon 24341, Republic of Korea; geodesy@kangwon.ac.kr (D.L.); qbkim1@kangwon.ac.kr (J.K.); 2 Department of Integrated Energy & Infra System, Kangwon National University, Chuncheon 24341, Republic of Korea; 3 Department of Ocean Civil Engineering, Gyeongsang National University, Tongyeong 53064, Republic of Korea

**Keywords:** sidewalk HD map, mobile LiDAR, pedestrian barrier, mobile mapping system (MMS), SLAM

## Abstract

Sidewalk high-definition (HD) maps require centimetre-level representation of pedestrian barriers to support mobility assistance and barrier-free infrastructure management. This study evaluates six mobile light detection and ranging (LiDAR) platforms for sidewalk HD mapping: terrestrial laser scanning (TLS), a push-cart mobile mapping system (MMS), two backpack systems (GNSS/INS (Global Navigation Satellite System/Inertial Navigation System)-aided and SLAM (simultaneous localization and mapping)-based), and two handheld systems (GNSS/INS-aided and SLAM-based). Surveys were conducted at two sites with contrasting occlusion and GNSS conditions (park and dense downtown corridors). Point clouds were transformed to a common control network, with independent checkpoints for absolute accuracy. The reference dataset achieved a planimetric root mean square error (RMSE) of 0.017–0.049 m and vertical RMSE of 0.009–0.014 m across sites. Platforms were compared for positional accuracy, point density, and extractability of key accessibility attributes (effective width, step height, and longitudinal slope). Cart-mounted MMS provided stable geometry under occlusion, while SLAM-based handheld mapping improved robustness in GNSS-degraded areas; backpack SLAM performance depended on loop-closure opportunities and scene dynamics. We provide guidance on selecting pedestrian-scale LiDAR platforms for sidewalk HD mapping under different survey conditions.

## 1. Introduction

### 1.1. Background and Motivation

National policies on barrier-free environments and mobility rights for transportation-disadvantaged users have recently been strengthened, which, in turn, has increased the demand for sidewalk high-definition (HD) maps capable of representing pedestrian environments in a detailed manner. Sidewalk width and the arrangement of green spaces and streetscape facilities significantly affect pedestrian satisfaction and preferences [1], and even where barrier-free design guidelines are applied, the current design criteria for pedestrian facilities often remain at a minimum level that is insufficient to ensure comfortable movement for mobility-impaired users [2,3].

Sidewalk HD maps can serve as foundational geospatial data commonly utilized in various applications, including pedestrian route guidance, mobility support for the transportation-disadvantaged, operational management of outdoor autonomous delivery or service robots, and maintenance of pedestrian infrastructure. International research has explored methods for automatically extracting sidewalk width, grade, and obstacle occupancy from mobile light detection and ranging (LiDAR), deep learning, and multimodal geospatial data, which have been applied to network-level sidewalk inventory construction, ADA-related accessibility assessment, derivation of effective walkable width, and pedestrian pathfinding [4,5,6,7,8,9]. For such applications, road centrelines alone are insufficient; pedestrian barriers—including sidewalk width, vertical steps, longitudinal grade, surface irregularities, tactile paving, and curb locations—must be represented in a stable and consistent manner at centimetre resolution.

Existing HD road maps have been produced mainly using vehicle-mounted mobile mapping systems (MMS), and therefore provide high-quality representations of carriageways and intersections. Prior work on HD map generation and localization for has established data models and workflows that accurately represent lanes, lane markings, road signs, and traffic signals using vehicle-based MMS [10,11,12,13,14,15]. In addition, the sensor configurations and processing pipelines of mobile mapping platforms have been comprehensively reviewed across different applications [16]. However, owing to the sensor vantage point and vehicle height, sidewalk sections frequently suffer from occlusions caused by parked vehicles, street trees, street furniture, awnings, and dense pedestrian flows. Vehicle-mounted LiDAR studies therefore often model sidewalk elements indirectly and within a limited lateral range along the driving trajectory [17,18]. Consequently, it is difficult to stably and exhaustively represent barrier elements across the full sidewalk width.

Recent studies have reported cost-effective LiDAR-based workflows for sidewalk evaluation and barrier detection (including 2D LiDAR scanners and portable SLAM/mobile mapping systems), highlighting the need for standardized, quantitative performance assessments that are linked to sidewalk data models [11,12,13]. If barrier-related attributes such as width, vertical steps, and grades are structurally encoded in the sidewalk network, barrier-free navigation services that selectively derive only those paths that are traversable by users with wheelchairs, strollers, or other mobility aids, as well as by outdoor service robots, can be realized. To enable this, acquiring dense 3D point clouds for a pedestrian space is not sufficient. A systematic framework is also required to consistently embed barrier thresholds as attributes in the sidewalk-related layers of HD road map data models such as RD_PATHWAY, PDST_LINK, PDST_NODE, and PDST_FACILITY. However, before the implementation of such a framework, it is necessary to quantitatively assess the combination of LiDAR platforms that satisfies the required accuracy, point density, occlusion recovery performance, and operational efficiency for sidewalk environments.

### 1.2. Objectives and Scope

The objective of this study is to quantitatively compare the performance of multiple LiDAR platforms that are potentially applicable to high-definition (HD) sidewalk map production, using test sites that reflect sidewalk-specific conditions. This work is positioned as a comparative performance evaluation and guideline-oriented study that supports equipment selection and deployment planning for sidewalk HD mapping, rather than as an algorithmic contribution. To this end, reference point clouds and a control network were established using a static terrestrial LiDAR scanner for two representative pedestrian environments: (i) an open park walkway and (ii) a neighborhood commercial street with mixed residential and school zones. Trolley-based MMS, backpack-type, and handheld LiDAR systems were then deployed along identical or comparable routes at each site.

The acquired point clouds were integrated into a common coordinate reference frame via global navigation satellite system (GNSS)/inertial navigation system (INS)-aided trajectory adjustment and simultaneous localization and mapping (SLAM)-based registration to the control network. Using the static terrestrial LiDAR point cloud as the ground-truth dataset, we compared geometric quality and positional accuracy across platforms and examined their applicability to sidewalk HD mapping.

The derived metrics and platform-specific characteristics were interpreted in relation to the attribute structures of sidewalk-related data model layers such as RD_PATHWAY, PDST_LINK, PDST_NODE, and PDST_FACILITY. Based on these results, we provide practical recommendations on (i) suitable platform combinations and deployment sections for sidewalk HD map production, and (ii) how barrier information can be embedded as stable, threshold-based attributes within the existing HD road map data model. The primary limitation of this study is that the evaluation is based on two representative Korean urban sidewalk environments; site-specific factors (e.g., pedestrian density, seasonal foliage, and GNSS conditions) may affect performance and should be considered when generalizing the findings.

## 2. Materials and Methods

### 2.1. Evaluation Procedure and Study Sites

The evaluation procedure in this study was designed to reflect typical sidewalk environments encountered in urban areas. Two test sections were selected to represent both open-sky sidewalks and densely built sidewalk corridors.

The first study site was a pedestrian promenade within the Jamsil Hangang Park area in Songpa-gu, Seoul, with a total route length of approximately 3.4 km (Table 1). This section is characterized by relatively wide sidewalks with a clear separation between pedestrian and bicycle flows and by continuous linear facilities such as curbs, tactile paving, and ramps. Along the route, various barrier candidates relevant to sidewalk HD mapping are present, including local changes in sidewalk width, stairways, and crosswalk approaches.

The second study site was a downtown sidewalk corridor in the vicinity of Gwanak Elementary School in Gwanyang-dong, Dongan-gu, Anyang-si, Gyeonggi-do, with a total route length of approximately 1.7 km (Table 1). This section represents a neighborhood commercial street environment with narrow effective sidewalks, frequent driveway connections, on-street parking, street trees, and overhanging signboards. Accordingly, it provides a suitable test bed for assessing LiDAR performance under severe occlusions and GNSS-degraded conditions.

**Figure 1:**
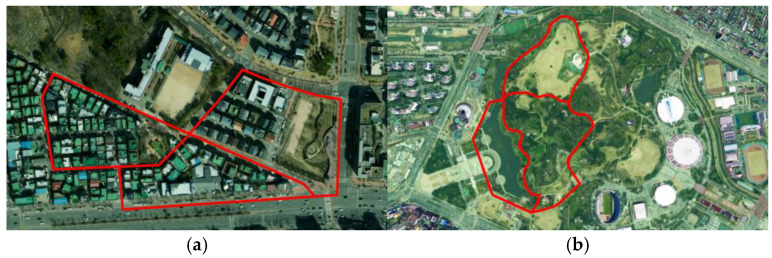
Study sites: (**a**) Park sidewalk in Seoul Olympic Park; (**b**) Downtown sidewalk near the Gwanak Elementary School (Anyang).

All data acquisitions were performed under daytime, clear-sky conditions (no precipitation) on dry pavement. Standard survey routes were planned to include key locations such as changes in sidewalk width, vertical discontinuities at curbs and ramps, longitudinal slope changes, stairways and ramps, segments with dense street furniture, and interfaces between sidewalks and carriageways. In several straight segments, static terrestrial LiDAR was deployed to establish reference point clouds and to support the construction of ground control points (GCPs), enabling verification of both positioning accuracy and registration performance of the mobile LiDAR platforms. The study-site layouts are illustrated in Figure 1.

Control points were surveyed at both sites to support georeferencing and independent accuracy assessment. A total of 24 control points were established across the two routes, consisting of 9 points on the park route and 15 points on the downtown route. Following a 1:5 hold-out rule, 2 points (park) and 3 points (downtown) were reserved as independent checkpoints (CPs) and were not used in any registration or adjustment step. The remaining points were used as GCPs for coordinate unification and strip/trajectory adjustment. Control points were distributed to cover the full route extent and key geometric changes (e.g., curves, intersections, and grade changes) while avoiding local concentration to reduce correlation and ensure objective validation [19,20]. In accordance with the Korean HD road-map regulation, control points were placed at stable, identifiable features in point clouds (e.g., pavement markings and crosswalk edges), with an average density of at least one point per kilometer and additional points in GNSS-degraded segments; checkpoints were allocated at a ratio of approximately one per five control points. Overall, the mean spacing was approximately 0.38 km for the Olympic Park route (9 points over 3.414 km) and 0.12 km for the Gwanak Elementary School corridor (15 points over 1.757 km).

### 2.2. Sensor Configuration and Survey Operation

In this study, a terrestrial static LiDAR system and five mobile LiDAR platforms were used. A terrestrial laser scanner Leica RTC360 (Leica Geosystems AG, Heerbrugg, Switzerland) was used as a reference instrument to establish the control network and reference point clouds within the test sections. Mobile platforms were categorized into cart-mounted mobile mapping systems (MMS) Leica Pegasus Two Ultimate (Leica Geosystems AG, Heerbrugg, Switzerland), backpack-type LiDAR systems Leica Pegasus Backpack (Leica Geosystems AG, Heer Brugg, Switzerland) and Map4 SEAMS ME (MAP IV, Inc., Tokyo, Japan), and handheld LiDAR systems Leica BLK2GO (Leica Geosystems AG, Heerbrugg, Switzerland) and CHCNAV RS10 (CHC Navigation, Shanghai, China).

The cart-mounted MMS was operated primarily along long sidewalk corridors to construct the centerlines of RD_PATHWAY and PDST_LINK, which formed the basic geometric framework of the sidewalk HD map. Backpack-type systems were assigned to supplement the observation gaps inside sidewalks where cart access is difficult, such as neighborhood commercial streets, narrow sidewalks, and internal sub-links. Handheld LiDAR systems have been used to capture detailed local geometries of barrier-concentrated segments, including curbs, tactile paving, stairs, and ramps, at close range. In practice, all platforms were planned to share the standard survey routes defined in the previous subsection so that the registration of their point clouds and a comparison of their performance could be carried out in a consistent manner. The configuration of the platforms and their role-sharing were determined with reference to previous studies that comprehensively summarized the sensor configurations, processing workflows, and application domains of vehicle-mounted, backpack-type, and handheld mobile mapping systems [16]

#### 2.2.1. Terrestrial LiDAR

For the terrestrial surveys, a Leica RTC360 was deployed at multiple scan stations along the test sections to construct a dense, high-accuracy reference point cloud. Scan stations were spaced to ensure at least 40% overlap between neighboring scans, and both artificial reflective targets and distinct natural features (e.g., edges of curbs, corners of walls, and poles) were used for registration between stations. The scanner was mounted on a tripod at approximately pedestrian eye level so that the geometry of the sidewalks, curbs, tactile paving, and barrier features could be captured in sufficient detail.

**Figure 2:**
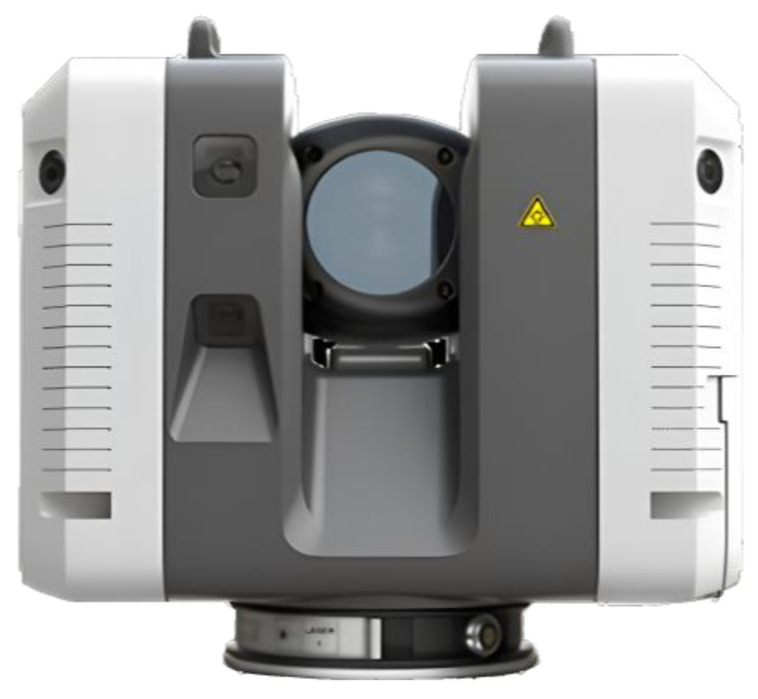
Leica RTC360.

A global network adjustment was performed using the GCPs measured by conventional surveying and scan station ties, minimizing the residuals between the control points and the registered point clouds. As a result, the combined standard deviation at independent check points was ±1.3 cm in planimetry and ±1.5 cm in elevation, which is sufficient to serve as a reference dataset for evaluating the mobile LiDAR data. Terrestrial LiDAR point clouds were used as (i) a reference control network and GCP coordinates, (ii) ground truth for assessing the absolute positioning accuracy of each platform, and (iii) reference data for deriving the threshold values for barrier extraction. The appearance and specifications of the terrestrial LiDAR system are summarized in Figure 2 and Table 2.

In an initial 70 m verification segment used to confirm reference integrity, five control points were measured (three correction points and two check points), and the terrestrial network adjustment achieved checkpoint standard deviations of ±1.3 cm (planimetry) and ±1.5 cm (elevation).

#### 2.2.2. Cart-Mounted Mobile Mapping System

The cart-mounted MMS integrates a GNSS receiver, an inertial measurement unit (IMU), and a rotating LiDAR sensor on a push cart adapted for sidewalk operations. The GNSS observations were corrected during post-processing using a post-processed kinematic (PPK) approach with reference data from continuously operating reference stations (CORS) operated by the National Geographic Information Institute. The GNSS and IMU data were combined to estimate the platform trajectory.

A GNSS antenna was installed at the top of the cart to maximize sky visibility, and the cart frame was designed to stably support the instrument body and batteries, while minimizing the vibrations transmitted to the sensors. Surveys were conducted along standard routes while maintaining an approximately constant walking speed and gentle changes in heading.

In the Olympic Park section, with an open-sky view, the focus was on acquiring long, continuous trajectories suitable for defining the centerlines of the RD_PATHWAY and PDST_LINK features in the sidewalk HD map. In the downtown section near Gwanak Elementary School, emphasis was placed on identifying observation gaps in front of building façades and within sidewalks, as well as on characterizing GNSS-degraded segments.

**Figure 3:**
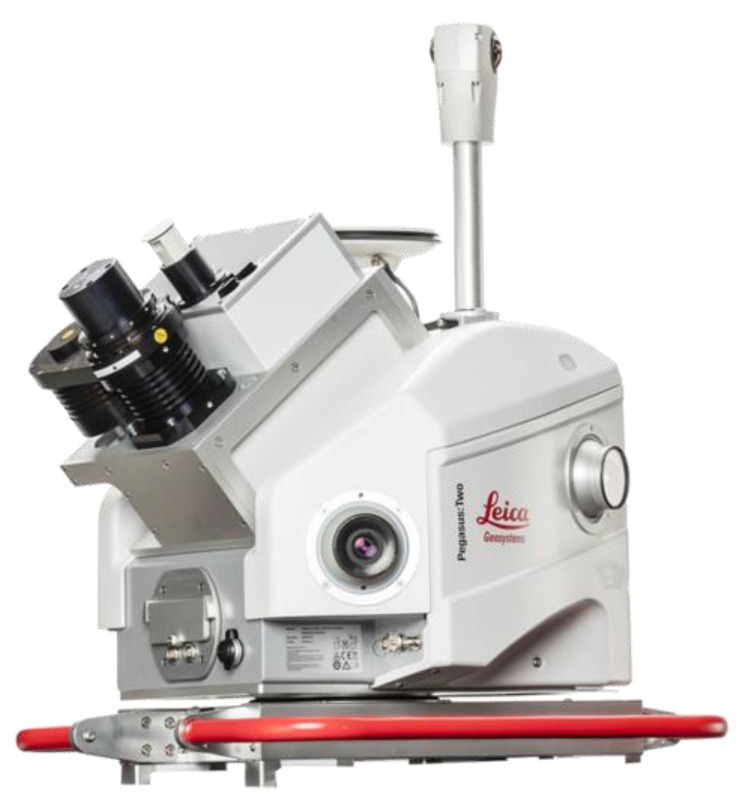
Leica Pegasus Two.

Raw LiDAR scans were georeferenced using the combined GNSS/IMU trajectory, and the residuals at the crossing trajectories were examined to minimize stripwise misalignments. The final outputs consisted of trajectory-wise point clouds (LAZ format), combined GNSS/IMU trajectories, lever arm and boresight parameters, post-processed positioning results, and strip-adjustment residual records. The sensor configuration and specifications of the cart-mounted MMS are presented in Figure 3 and Table 3, respectively.

#### 2.2.3. Backpack-Type LiDAR

Backpack-type LiDAR systems acquire data while the operator walks along the sidewalk wearing a LiDAR unit on the back. This platform is suitable for the continuous surveying of segments where cart access is difficult, such as narrow sidewalks, stairways, pedestrian overpasses, and storefront corridors [21,22,23,24].

In this study, backpack systems comprising a GNSS antenna and receiver, an IMU, and a rotating LiDAR sensor were used. Trajectories were estimated by combining the GNSS and IMU data, and LiDAR-based simultaneous localization and mapping (SLAM) was applied in parallel. Real-time kinematic (RTK) corrections from nearby control points were applied to the GNSS to secure an absolute positioning reference, whereas the raw data recorded during acquisition were used in postprocessing for quality control and consistency checks of the corrections.

**Figure 4:**
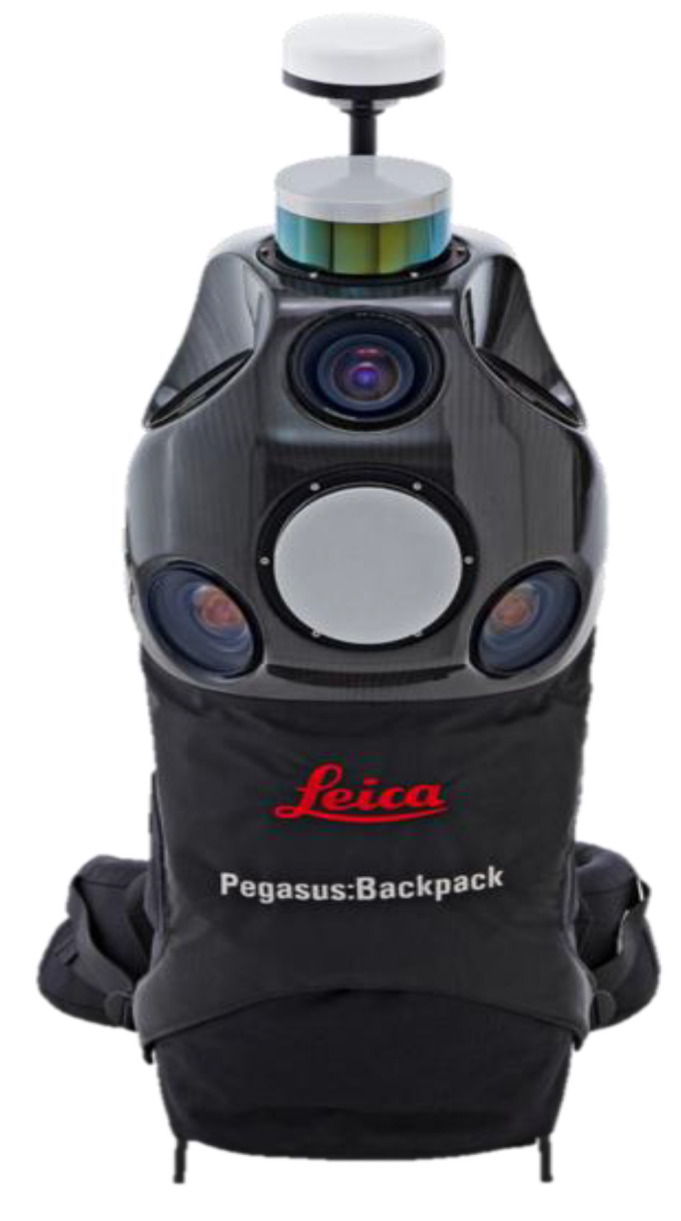
Pegasus Backpack.

**Figure 5:**
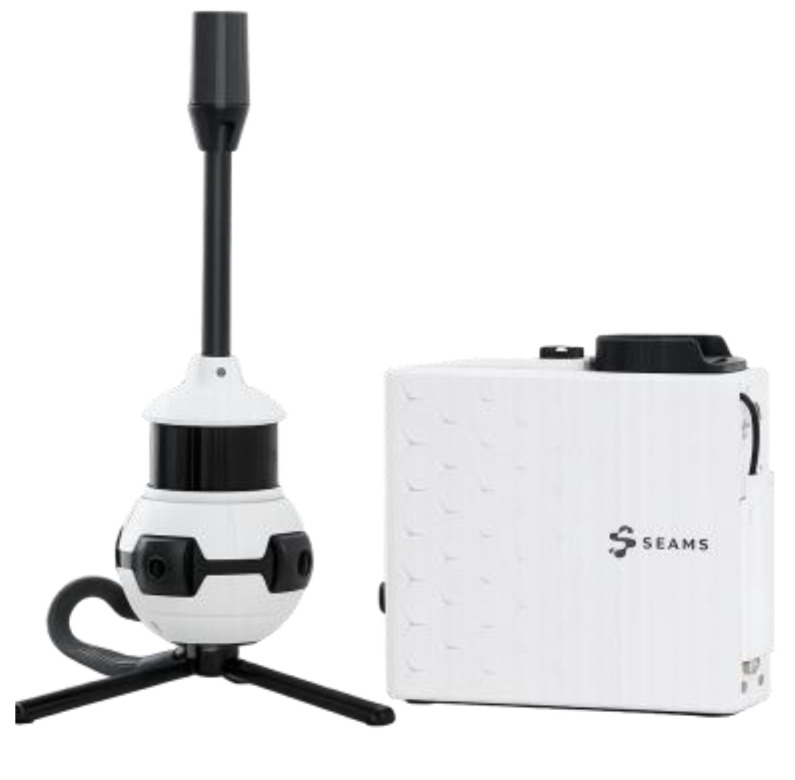
Map4 SEAMS ME.

The walking paths were planned primarily as loops in which the start and end points coincided, and additional crossing loops were included where appropriate to improve the stability of the SLAM trajectories. In sections with vertical discontinuities, such as stairs, thresholds, and curbs, the operator followed linear and planar features, such as walls and columns; thus, feature-based registration could be performed robustly. The appearance and specifications of the backpack-type LiDAR systems are provided in Figure 4 and Figure 5 and Table 4 and Table 5.

#### 2.2.4. Handheld LiDAR

Handheld LiDAR systems operate by manually sweeping the scanner while walking, allowing flexible data acquisition in confined spaces and complex geometries. In this study, the Leica BLK2GO and CHCNAV RS10 were used as representative handheld platforms.

Both systems are based on LiDAR SLAM without GNSS and therefore rely on loop closures and feature-based matching for trajectory estimation. Survey routes were designed to include frequent loop closures and minimize segments with monotonous geometry, where SLAM drift could become significant. In sections with dense street furniture or interior-like sidewalk spaces, the operator traced walls, columns, and other structural features to stabilize the SLAM solution [20,25,26,27,28,29,30,31,32,33,34].

**Figure 6:**
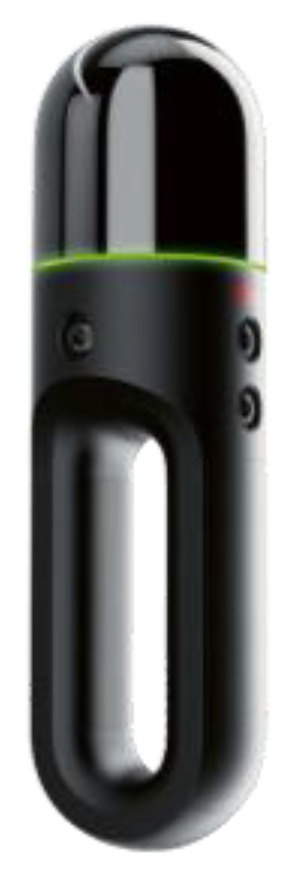
Leica BLK2GO.

**Figure 7:**
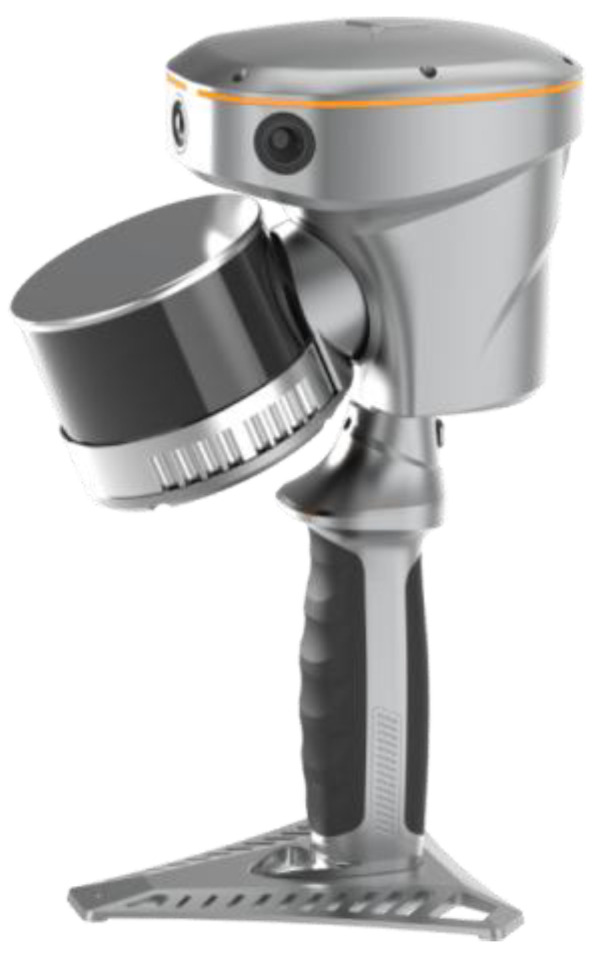
CHCNAV RS10.

To connect the handheld LiDAR point clouds to a common control framework, artificial targets and natural feature points commonly visible in the terrestrial and mobile datasets were used as tie points. These constraints were then incorporated into the subsequent multi-platform registration step. The configurations and specifications of the handheld LiDAR systems are summarized in Figure 6 and Figure 7 and Table 6 and Table 7.

### 2.3. Data Processing and Registration Procedure

The data processing workflow in this study was designed to integrate multi-platform LiDAR datasets into a unified project coordinate system and to provide stable reference data for evaluating sidewalk HD map performance. The procedure consists of four main stages: (i) temporal alignment, (ii) coordinate unification and initial registration, (iii) quality control and strip adjustment, and (iv) registration to the reference dataset and inter-platform fusion [19,20,21,22,23,24,35,36].

The project-standard coordinate system was defined by VRS-based GNSS surveying as UTM Zone 52N with ellipsoidal heights, and all platform point clouds and trajectories were transformed and evaluated in this common reference frame.

In the temporal alignment stage, the internal time stamps of each sensor were checked against the GNSS time and terrestrial LiDAR acquisition times, and all raw data streams were synchronized to a common time axis. For mobile platforms, GNSS/INS integration or SLAM-based relative trajectories were computed, and scan-time tags were assigned to each LiDAR point based on the corresponding trajectory solution. Terrestrial LiDAR data were processed through station-to-station registration to form a consistent project coordinate system.

Second, in the coordinate unification and initial registration stages, the control network and ground control points (GCPs) established from the terrestrial LiDAR were used to transform each platform’s point cloud into a project-standard coordinate system. The mobile platform point clouds were first converted from sensor coordinates to ground coordinates using platform trajectories, lever arms, and boresight parameters. The initial rigid body transformations were then estimated in overlapping areas using terrestrial reference point clouds. Fine registration was performed using an iterative closest point (ICP) approach with point-to-plane constraints, and registration quality was summarized using cloud-to-cloud distance statistics (mean, RMSE, and selected percentiles) within overlapping sidewalk surfaces and curb planes. The allowable combined error thresholds were set to 0.03 m in both the horizontal and vertical directions, based on the coordinate differences at the control and checkpoints. For sessions that did not satisfy these criteria, the transformation parameters were re-estimated, problematic subsections were segmented, and survey sessions were re-selected.

Third, in the quality control and strip adjustment stages, the vertical and lateral residuals between the strips and the crossing trajectories were examined for each platform. LiDAR points corresponding to moving objects and obvious noise were first removed using intensity and incidence angle information. Strip adjustment was then performed by enforcing consistency along the linear and planar features such as the sidewalk edges, curbs, and building corners. After strip adjustment, iterative corrections were applied until the vertical differences at crossing segments were within 0.02 m and local lateral deviations were within 0.03 m. Sections that still failed to meet these criteria were excluded from subsequent performance evaluations. This process stabilized the relative geometric consistency of the point clouds from each platform.

Summary of key criteria and thresholds used in processing: (i) Initial registration and coordinate transformation were accepted when both horizontal and vertical residuals at control/check points were within 0.03 m; otherwise, transformation parameters were re-estimated and problematic subsections were segmented and reprocessed. (ii) Strip adjustment was iterated until vertical differences at crossing segments were within 0.02 m and local lateral deviations were within 0.03 m. (iii) Survey sessions that still failed to meet these criteria after reprocessing were excluded from subsequent performance evaluation.

Fourth, in the registration-to-reference and interplatform fusion stages, terrestrial LiDAR reference point clouds, cart-mounted MMS data, and backpack-type LiDAR data were used as primary references. Interplatform registration was performed using a combination of point constraints at the corners, columns, and curb steps, together with planar-consistency constraints. The transformation parameters and residuals obtained during the registration process were stored as session-level metadata, and the means, standard deviations, and maximum vertical differences relative to the reference point clouds were computed. In cases where the vertical RMSE exceeded 0.03 m or where pronounced spatial trends in residuals were observed in certain areas, the SLAM trajectories and registration constraints were adjusted to correct low-frequency distortions [37,38,39,40].

**Figure 8:**
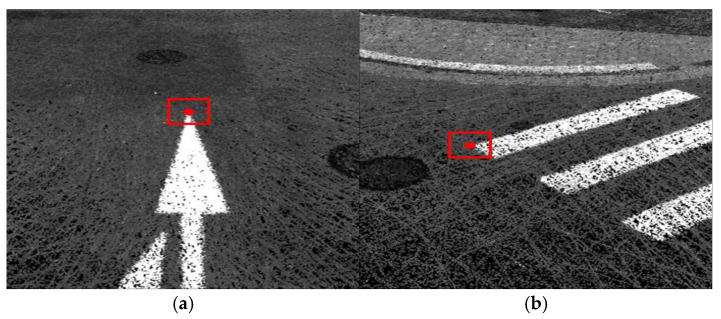
Examples of selecting ground control features: (**a**) road marking; (**b**) crosswalk. The red square indicates the selected feature region, and the white circle indicates the selected ground control feature point.

**Figure 9:**
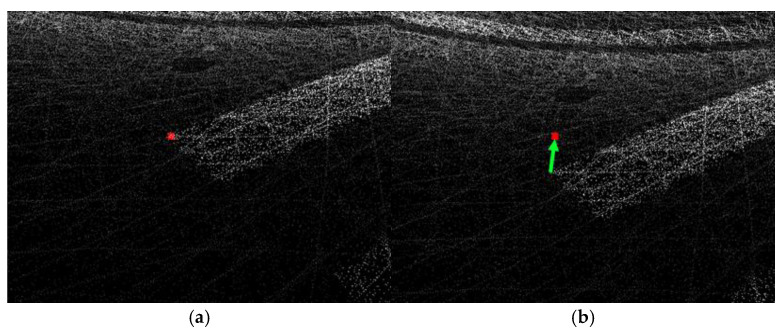
Correction of point-cloud data using ground-control results: (**a**) point cloud after control point correction; (**b**) point cloud before control point correction. The red circle marks the selected ground control feature point, and the green arrow highlights the corresponding area of interest.

Finally, an integrated point cloud aligned to the terrestrial LiDAR control network is generated, and the point clouds from the cart-mounted MMS, backpack-type, and handheld platforms are compared and evaluated within a common horizontal coordinate system and vertical datum. This integrated point cloud served as the basis for computing evaluation indicators such as positioning and registration accuracy, point density, continuity of coverage, and occlusion-recovery performance. It also provides an underlying dataset for analyzing the feasibility of extracting barrier-related attributes in conjunction with the RD_PATHWAY, PDST_LINK, PDST_NODE, and PDST_FACILITY data models. Examples of the GCP selection and correction processing are shown in Figure 8 and Figure 9, respectively.

### 2.4. Definition of Evaluation Items and Indicators

The performance of each LiDAR platform was evaluated from the perspective of sidewalk HD map production by focusing on the following indicators: positioning and registration accuracy, point density and coverage continuity, occlusion recovery performance, work efficiency and safety, and linkages to the sidewalk data model.

First, positioning accuracy was assessed using horizontal and vertical residual statistics at independent check points computed against the terrestrial reference dataset. For each platform, the mean error, standard deviation, root mean square error (RMSE), and maximum deviation were derived for the planimetric and vertical components. To keep the evaluation criteria consistent with the formal project standards, results were interpreted with respect to the national point-cloud positional-accuracy requirements (absolute accuracy: ≤0.2 m in both planimetry and elevation, with maximum errors ≤ 0.4 m). For relative alignment between overlapping point clouds (e.g., inter-session strip alignment or integration with adjacent datasets), we additionally report RMSE-based 95% confidence intervals computed as 1.7308 × RMSE_xy for planar accuracy and 1.9600 × RMSE_z for vertical accuracy, and compare them to the MMS alignment criterion (95% CI ≤ 0.1 m in both planimetry and elevation; maximum errors ≤ 0.2 m). Residuals obtained during inter-platform registration were used as supplementary indicators of relative registration quality.

The point density and continuity of coverage were evaluated by defining a regular grid and calculating the mean and minimum number of points per cell, as well as the proportion of cells containing at least one point. This analysis was used to confirm whether sufficient point-cloud resolution was secured around candidate barrier locations, such as sidewalk edges, curbs, tactile paving, and steps, and whether continuous point clouds without gaps were obtained along extended pedestrian routes.

Occlusion recovery performance was examined in sections with major occluding objects such as parked vehicles, trees, roadside furniture, and pedestrian clusters. For these sections, we evaluated whether barrier attributes such as effective width, level difference, and longitudinal slope could be derived from each platform’s point cloud and whether the derived values were mutually consistent. For identical test segments, the differences in the extracted width, step height, and slope among the platforms were compared, and segments in which barrier attributes could not be stably estimated from a given platform were identified to clarify platform-specific limitations.

Work efficiency and safety were evaluated using indicators such as observable distance per unit time; time required for equipment setup, removal, and relocation; required number of operators; and degree of interference with pedestrians and vehicular traffic along the survey routes. These indicators were used to compare the deployment scale and on-site burden associated with each platform while acquiring data of comparable quality.

Finally, the data-model linkage was evaluated for the main sidewalk HD map feature classes, namely RD_PATHWAY, PDST_LINK, PDST_NODE, and PDST_FACILITY. For each data model object, we summarized the geometric information and barrier attributes that could be provided by each platform and examined whether they satisfied the threshold values and accuracy requirements assumed in the data model. On this basis, we analyzed the extent to which each platform and platform combination could reliably provide barrier-related attributes, such as effective width, level difference, longitudinal gradient, and surface condition, and derived suitable configurations and operational strategies for sidewalk HD map production.

## 3. Results of Platform Performance Comparison

Survey conditions and processing thresholds. All acquisitions were conducted during daylight hours under clear-sky conditions with dry pavement, as documented in the field log. Survey dates and acquisition time windows (local time and UTC) are summarized in Table 1. For reproducibility, the key registration and strip-adjustment acceptance thresholds are described in Section 2.3.

### 3.1. Segment-Based Performance Comparison

#### 3.1.1. Comparison of Positional Accuracy

The positioning accuracy of each platform was evaluated with respect to the reference point cloud and control points acquired from the static terrestrial LiDAR survey. The reference dataset achieved a composite error on the order of a few centimeters in both the planimetric and vertical directions at the checkpoints and was therefore considered sufficiently accurate to serve as the ground truth for subsequent comparison.

The cart-based MMS exhibited horizontal errors of several centimeters and vertical errors of a few centimeters in the open Olympic Park segment, with planimetric and height differences relative to the reference data distributed relatively uniformly along the trajectory. However, in the dense old-town segment, the partial deterioration of the GNSS reception and multipath effects caused local bias and increased scatter, particularly near street trees, parked vehicles, and building façades.

The backpack-type MMS showed comparable or slightly larger errors than the cart-based MMS in the park segment; however, its positioning accuracy degraded more severely in the old-town environment, where GNSS visibility was restricted and frequent heading changes occurred. The handheld LiDAR platform, which relies primarily on SLAM without direct GNSS integration, yielded a relatively stable internal geometry but exhibited global offsets when loop-closure constraints were insufficient or when the trajectory length increased without revisit.

Notably, the SLAM-based handheld LiDAR achieved better absolute accuracy than the GNSS/INS-aided handheld unit, which is consistent with the positive role of SLAM in constraining short pedestrian trajectories. In contrast, the SLAM-based backpack system did not always outperform the GNSS/INS-aided backpack system. This outcome can be explained by (i) longer trajectory lengths and fewer closed loops for the backpack route, which reduce the strength of loop-closure constraints; (ii) increased dynamic objects (pedestrians and vehicles) and repeated façade patterns that weaken scan-to-scan matching; and (iii) residual heading drift accumulating during segments with limited geometric features (e.g., long straight corridors) before revisiting previously mapped areas. Meanwhile, the GNSS/INS-aided backpack solution benefited from absolute trajectory constraints and subsequent control-point-based correction, which reduced global offsets even when local SLAM consistency was high. These factors indicate that SLAM is not uniformly beneficial; its impact depends on loop-closure opportunities, scene structure, and the balance between local registration and absolute georeferencing.

The quantitative statistics of the horizontal and vertical errors for each platform and segment are summarized in Table 8, using the RMSExy, RMSEz, 95th-percentile horizontal distance, 95th-percentile height error, and maximum error as evaluation indicators. As summarized in Table 8, the cart-based MMS generally achieves the smallest RMSExy and RMSEz in the park segment, whereas the performance gap between the platforms increases in the more challenging old-town segment. From a sidewalk HD map perspective, the results in Table 8 indicate that all mobile platforms can satisfy the 0.2 m point-cloud accuracy requirement in the park-type environment, whereas only the cart-based MMS consistently meets the same criterion in the old-town segment without additional control or correction measures.

#### 3.1.2. Point Density and Continuity of Coverage

Point density and continuity of coverage were evaluated along standard observation routes for both segments. For each platform, the number of LiDAR points within a unit area was computed on the cross-sections and along the centerline of the sidewalk, and gaps in coverage were identified where the point density fell below the minimum threshold required for reliable feature extraction.

The cart-based MMS provided the highest and most uniform point density throughout both segments owing to its stable sensor platform and constant scanning geometry. In the park segment, even the curb edges, ramps, and changes in the longitudinal grade were represented by dense point clusters, enabling robust extraction of the centerline, edge lines, and barrier features. In the old-town segment, the point density locally decreased near parked vehicles and street furniture; however, the cart platform maintained continuous coverage along most of the effective walking corridors.

The backpack-type MMS achieved sufficient point density along the main walking path, but showed partial thinning of points on the outer edge of the sidewalk and near façade side obstacles owing to the operator’s walking trajectory and body occlusions. The handheld LiDAR system exhibited the largest variability in density, with very high local densities near the operator and occasional gaps or sparse regions where the scanning direction was not aligned with narrow passages or where the SLAM drift caused misregistration.

These results highlight that even when the positioning accuracy is comparable across platforms, the point density and continuity of coverage can become limiting factors for sidewalk HD map construction on complex urban streets. In particular, the cart-based MMS secured the most stable point density and continuous coverage along the main walking corridors, whereas the backpack-type MMS and handheld LiDAR tended to exhibit larger local variations in density and more frequent gaps around occluding objects and narrow passages.

#### 3.1.3. Barrier Extraction and Occlusion Recovery Performance

The barrier extraction and occlusion recovery performance were assessed in sections where occluding objects such as parked vehicles, trees, street furniture, and pedestrian clusters were present. For each platform, we evaluated [1] whether key barrier attributes—sidewalk width, step height at curb and ramps, and longitudinal slope—could be computed and [2] whether the derived values remained consistent with those obtained from the reference dataset. A quantitative comparison of representative curb height and sidewalk width measurements against TLS ground truth is provided in Table 9. In addition, the platform-dependent surface point density under open-sky and occluded/downtown conditions is summarized in Table 10, and the longitudinal grade estimation performance and pass/fail results under the ±1.0% criterion are reported in Table 11.

In the park segment, most barrier attributes were stably extracted from all platforms, with minor discrepancies in the local step height and slope values. However, in the old-town segment, the cart-based MMS was the only platform that consistently preserved sufficient geometry behind parked vehicles and near façade-side obstacles to allow reliable estimation of sidewalk width and longitudinal grade. The backpack-type MMS sometimes failed to resolve the full cross-sectional profile in narrow sidewalks owing to body- and trajectory-induced occlusions, whereas the handheld LiDAR frequently produced incomplete curbs and distorted step geometry when SLAM drift occurred in highly cluttered areas.

#### 3.1.4. Work Productivity and On-Site Safety

Work productivity and on-site safety were compared using indicators such as survey speed (km/h), net observable length per unit time, time required for equipment setup/removal/relocation, required number of operators, degree of interference with pedestrian flow and adjacent traffic, and practical constraints under narrow-sidewalk conditions. In addition, economic and operational feasibility were discussed qualitatively in terms of capital cost class (high/medium/low), consumables and maintenance burden, and the marginal cost of adding supplementary surveys to address occlusion-induced gaps.

In the park segment, the cart-based MMS demonstrated the highest productivity for long, continuous corridor mapping because the platform can travel at a nearly constant speed on a relatively flat surface while maintaining stable sensor geometry and GNSS visibility. The required crew size was typically two (operator and safety spotter), and the workflow yielded trajectory-consistent datasets well suited to defining RD_PATHWAY and PDST_LINK centerlines and boundaries with minimal rework.

In the old town segment, the productivity of the cart-based MMS decreased owing to intermittent stops, detours around obstacles, and the need to share narrow passages with pedestrians. Nevertheless, the cart platform remained advantageous for generating a consistent link-level geometric backbone, particularly when supplemented with short stationary pauses at critical occlusion hotspots to improve coverage behind parked vehicles and street furniture.

The backpack-type MMS provided high walking-speed flexibility and reduced interference with pedestrians in confined environments, enabling efficient coverage of sub-links and façade-side corridors where cart access is limited. However, operator-body occlusions and frequent heading changes increased the likelihood of localized coverage gaps, which can lead to additional re-surveys or post-processing effort. From an economic perspective, backpack systems generally offer a medium capital-cost class with a favorable balance between mobility and data quality when GNSS reception is partially degraded but sufficient loop geometry is available for SLAM stabilization.

Handheld LiDAR was the most flexible for close-range acquisition at barrier-concentrated locations (e.g., curbs, tactile paving, stairs, and ramps) and can be operated by a single person with minimal setup time and low disruption. Its principal limitation is the higher risk of global distortion or incomplete geometry in long, monotonous segments without reliable loop closures, which can increase the alignment effort to the control network. The overall platform suitability and barrier-related performance considered in this productivity and safety discussion are summarized in Table 12. Overall, the results indicate that for typical sidewalk HD mapping, a cart-based MMS should be used to establish the primary link geometry, while backpack and handheld platforms are best deployed as complementary tools to recover occluded or detailed barrier geometries in targeted segments; this combination provides the most favorable balance among data quality, work efficiency, and practical operating cost.

### 3.2. Applicability to Sidewalk HD Map Construction

#### 3.2.1. Applicability for RD_PATHWAY and PDST_LINK

RD_PATHWAY and PDST_LINK are the core data model layers that represent the centerlines and link structures of the pedestrian networks in the HD road map. Their key requirements include the long-range continuity of geometry, positional accuracy at the HD map level, and global consistency with existing road networks.

Applying the positioning and density/coverage indicators presented in Section 3.1, the cart-mounted MMS was found to satisfy the 0.2 m point-cloud accuracy criterion in both the park-type and old-town segments, while maintaining the most stable point density and coverage continuity along the standard survey routes. In the Olympic Park section, the cart-mounted MMS provides long, continuous trajectories that are well-suited for defining the RD_PATHWAY and PDST_LINK centerlines. The neighborhood street section near Gwanak Elementary School also secures sufficient geometric continuity behind occluding objects, such as parked vehicles and street trees, enabling the reconstruction of link-level structures, even under challenging GNSS and occlusion conditions.

Backpack-type LiDAR systems can capture centerlines that follow actual pedestrian paths more closely and are particularly effective in segments where cart access is difficult, such as stairways, pedestrian-only alleys, and interior link connections. However, because the absolute position of the backpack trajectories is more sensitive to GNSS degradation and SLAM stability, their centerlines are the most reliable when anchored to the cart-mounted MMS and terrestrial reference data. Therefore, backpack systems are better used as auxiliary platforms that supplement and refine MMS-based RD_PATHWAY and PDST_LINK networks, rather than as the sole source of link geometry.

Handheld LiDAR is mainly used to inspect and locally refine the geometry of link junctions and access points, including stairs, ramps, and narrow passages, rather than constructing long-range centerlines on its own. In practice, handheld point clouds are utilized to validate and adjust the connectivity between the RD_PATHWAY and PDST_LINK elements at entrances, stair/ramp connections, and narrow access points, where MMS and backpack data alone are insufficient to confirm actual traversability.

In summary, for the RD_PATHWAY and PDST_LINK constructions, the cart-mounted MMS is the primary platform that provides long-range, accurate, and continuous centerlines, whereas the backpack and handheld LiDAR systems play complementary roles by filling gaps and refining local connectivity in areas where the cart platform is constrained.

#### 3.2.2. Applicability for PDST_NODE, PDST_FACILITY and Barrier Representation

PDST_NODE and PDST_FACILITY represent the nodes and facility elements in a pedestrian environment such as crosswalks, intersections, entrances, stairs, ramps, tactile paving, elevators, and escalators. These elements are directly related to barrier attributes, including the effective width, vertical steps, longitudinal gradient, and surface roughness, which are crucial for sidewalk HD maps aimed at accessibility analysis and robot navigation.

In this study, terrestrial LiDAR was used to establish reference geometries for representative PDST_NODE and PDST_FACILITY features within the test sections and to set threshold values for barrier attributes such as curb height, stair riser height, and ramp gradient. When evaluated against these thresholds, the backpack-type and handheld LiDAR systems proved particularly advantageous for extracting detailed barrier attributes at the node and facility levels. Because backpack systems follow pedestrian routes, they can continuously capture the geometry of the crosswalk steps, ramp start and end points, tactile paving zones, and entrance thresholds around the PDST-NODE locations. Handheld LiDAR provides a very high point density in the immediate vicinity of barrier elements, enabling the centimeter-level derivation of step heights, gradients, and effective widths for PDST_FACILITY objects, such as stairs, ramps, and curb ramps.

The cart-mounted MMS, while excellent for link-level structure and for locating the general positions of nodes and facilities, has relative limitations in representing fine-scale geometries, such as subtle level differences at sidewalk–road interfaces and detailed tactile paving patterns, especially under strong occlusion. Consequently, the most efficient strategy for PDST_NODE and PDST_FACILITY mapping is to use terrestrial, backpack, and handheld LiDAR as primary data sources for barrier attributes and to use the cart-mounted MMS as a structural backbone that provides the network context and global positioning into which these detailed datasets are integrated.

The platform roles and recommended combinations for different sidewalk environments are summarized in Table 13, which outlines the primary and supplementary platforms, expected benefits, and implementation notes by environment type. As Table 13 indicates, a configuration centered on a cart-mounted MMS reinforced by a backpack-type MMS and handheld LiDAR in locally constrained or highly occluded sections provides a practical and robust deployment strategy for sidewalk HD map production. This division of roles allows the positional accuracy, point density, barrier representation capability, work efficiency, and on-site safety to be balanced while aligning directly with the attribute requirements of the RD_PATHWAY, PDST_LINK, PDST_NODE, and PDST_FACILITY data models.

## 4. Conclusions

This study conducted a comparative performance evaluation of multiple LiDAR platforms applicable to high-definition (HD) sidewalk map production, focusing on test sections that represented the specific constraints of pedestrian spaces. Static terrestrial LiDAR was used to establish a control network and reference point cloud in two contrasting environments: a park-type walkway in an urban park and a mixed-use everyday street with shops and a primary school. Cart-mounted MMS, backpack, and handheld systems were deployed along common or overlapping survey routes to generate integrated point clouds, which were evaluated in terms of positional and registration accuracy, point density and continuity of coverage, occlusion recovery performance, field productivity and safety, and consistency with the sidewalk data model.

In terms of positional accuracy and strip-adjustment quality, the cart-based MMS and SLAM-based backpack systems satisfied the tolerance required for HD sidewalk map production at the two test sites. The cart-based MMS achieved sufficient horizontal and vertical accuracies and stable strip adjustment for link-level centerline construction in both park-type and mixed-use sidewalk sections, indicating that it is suitable as a primary sensor for constructing the RD_PATHWAY and PDST_LINK frameworks.

The backpack LiDAR, which combines GNSS and SLAM, enabled near-walking-speed continuous mapping even in GNSS-denied segments and provided superior continuity in narrow sidewalks and highly curved pedestrian corridors. However, trajectory instability and degraded positional accuracy occurred when sharp direction changes and weak connections to high-precision control networks were present. Therefore, the backpack system was more efficient when used not for RD_PATHWAY and PDST_LINK centerline construction but to complement PDST_NODE and PDST_FACILITY objects and barrier attributes such as tactile paving, ramps, and step edges.

Handheld LiDAR proved effective for high-resolution close-range mapping at local barrier locations such as building entrances, steps and ramps, tactile paving, and curbs. Its point clouds allowed the direct estimation of attributes such as step height, longitudinal slope, and effective width at the node or facility level with centimeter-level precision. In contrast, the cart-based MMS was advantageous for capturing the overall link geometry and surrounding street furniture but had limitations in representing fine-scale barriers such as detailed tactile patterns or small discontinuities inside the sidewalk. Consequently, point clouds acquired with static terrestrial LiDAR, backpack, and handheld platforms should be used as the primary data source for PDST_NODE, PDST_FACILITY, and barrier attributes, whereas cart-based MMS provides a higher-level structural context and connectivity.

Synthesizing these findings, the most effective configuration for sidewalk HD map production is to use the cart-based MMS as the backbone sensor to construct RD_PATHWAY and PDST_LINK and to use backpack and handheld LiDAR to enrich PDST_NODE, PDST_FACILITY, and barrier attributes. This division of roles allows the balanced achievement of positional accuracy, point density, level of detail in barrier representation, and field productivity and safety while directly linking the outcomes of sensor performance to the requirements of the sidewalk data model. By explicitly relating each platform to the RD_PATHWAY, PDST_LINK, PDST_NODE, and PDST_FACILITY objects, the study organized the coverage and limitations of barrier attributes that can be derived from each point cloud and discussed the applicable segments and role sharing for different sensor combinations. The operational implications, including the expected benefits and cautions for each combination, are summarized in Table 13.

Table 13 also consolidates the capability of each platform to meet the common numerical thresholds adopted in this study—2 cm for step detection and ±1% for slope error—together with the positional-accuracy requirement and the level of detail needed for barrier-attribute mapping. Overall, the cart-based MMS exhibited the most consistent performance across the evaluated criteria, whereas the backpack-type MMS and handheld LiDAR more often showed conditional satisfaction in segments with severe occlusion and GNSS-degraded conditions.

This study has several limitations that should be considered when interpreting the results. First, the experiments were conducted at two representative Korean sidewalk environments; performance may vary under different urban morphologies, pedestrian densities, seasonal foliage conditions, and GNSS/Multipath environments. Second, the reference dataset was constructed using static terrestrial LiDAR and a control network; although checkpoint residuals indicate centimeter-level consistency, residual systematic effects may remain in highly occluded areas. Third, handheld and SLAM-dependent platforms are sensitive to route design (loop closures) and scene texture; therefore, the reported performance should be understood as achievable under the designed acquisition protocol rather than as an unconditional guarantee. Future work will expand the evaluation to additional cities and conditions and will refine standardized field protocols and data-model thresholds for barrier attributes to support scalable sidewalk HD map production.

## Figures and Tables

**Table 1:** Overview of study sites.

ID	Section Name	Location	Length
1	Park-road sidewalk inside Olympic Park	424, Olympic-ro, Songpa-gu, Seoul	3.4 km
2	Downtown sidewalk near Gwanak Elementary School	Around 1733-7, Gwanyang-dong, Dongan-gu, Anyang-si	1.7 km

**Table 2:** Leica RTC360.

Acquisition Mode	Static (Tripod)
SLAM	Not applied
GNSS	Not equipped
Correction	GCP
LiDAR rate (pts/s)	2,000,000
Channels	1
Range (m)	0.5–130
Fine accuracy	0.1 cm
Cameras	-
Coverage (FOV)	-

**Table 3:** Leica Pegasus Two Ultimate.

Acquisition Mode	Cart-Mounted Mobile Mapping
SLAM	Not applied
GNSS	Equipped
Correction	PPK
LiDAR rate (pts/s)	1,000,000
Channels	1
Range (m)	0–119
Fine accuracy	1–2 cm
Cameras	4 × 4000 × 3000
Coverage (FOV)	360°

**Table 4:** Pegasus Backpack.

Acquisition Mode	Backpack (Pedestrian)
SLAM	Not applied
GNSS	Equipped
Correction	PPK
LiDAR rate (pts/s)	600,000/300,000
Channels	16/32
Range (m)	0–100
Fine accuracy	2–3 cm
Cameras	5 × 2046 × 2046
Coverage (FOV)	360°

**Table 5:** Map4 SEAMS ME.

Acquisition Mode	Backpack (Pedestrian)
SLAM	Applied
GNSS	Equipped
Correction	RTK/PPK
LiDAR rate (pts/s)	640,000
Channels	32
Range (m)	0.3–120
Fine accuracy	1 cm
Cameras	4 × 1920×1280
Coverage (FOV)	360°

**Table 6:** Leica BLK2GO.

Acquisition Mode	Handheld (Near-Field)
SLAM	Not applied
GNSS	Not equipped
Correction	GCP/external alignment
LiDAR rate (pts/s)	420,000
Channels	1
Range (m)	0.5–25
Fine accuracy	1 cm
Cameras	3 × 4096 × 2048
Coverage (FOV)	360°

**Table 7:** CHCNAV RS10.

Acquisition Mode	Handheld (Pedestrian)
SLAM	Applied
GNSS	Equipped
Correction	RTK/PPK
LiDAR rate (pts/s)	320,000
Channels	16
Range (m)	0.05–120
Fine accuracy	1 cm
Cameras	3 × 2592 × 1944
Coverage (FOV)	270°

**Table 8:** Positional accuracy of each platform (CI: confidence interval). (XY) denotes horizontal (planimetric) accuracy, and (Z) denotes vertical accuracy.

Platform	RMSE (XY)	RMSE (Z)	95% CI (XY)	95% CI (Z)	Absolute Accuracy (≤0.2 m)	MMS Alignment (95% CI ≤ 0.1 m)
Cart-mounted MMS	0.045	0.012	0.078	0.024	Yes	Yes
Backpack LiDAR	0.020	0.020	0.035	0.039	Yes	Yes
SLAM-based backpack	0.042	0.021	0.073	0.041	Yes	Yes
Handheld LiDAR	0.091	0.079	0.158	0.155	Yes	No
SLAM-based handheld	0.020	0.009	0.035	0.018	Yes	Yes

**Table 9:** Quantitative comparison of sidewalk width against TLS ground truth.

Platform	Measured Width	TLS (GT)	Δabs (= |Platform − TLS|)
TLS (Ground Truth)	3.090	3.090	0.000
Cart-mounted MMS	3.126	3.090	0.036
Backpack LiDAR	3.128	3.090	0.038
SLAM-based backpack	3.123	3.090	0.033
Handheld LiDAR	3.136	3.090	0.046
SLAM-based handheld	3.089	3.090	0.001

**Table 10:** Surface point density by platform.

Platform	LiDAR Rate (pts/s)	(PDI_{Max}) (pts/m^2^)	Surface Density (Open Sky) (pts/m^2^)	Surface Density (Occluded/Downtown) (pts/m^2^)
Terrestrial LiDAR	1,802,347	409,612	3217	1893
Cart-mounted MMS	903,581	121,437	1963	1241
Backpack LiDAR	552,904	16,732	4786	2917
SLAM-based backpack	618,429	22,389	6143	3712
Handheld LiDAR	383,116	9764	2287	1463
SLAM-based handheld	312,578	14,183	3024	1821

**Table 11:** Longitudinal grade estimation results.

Platform	Estimated Grade (%)	Error vs. 5.0% (% *p*)	Absolute Error (% *p*)	Pass (±1.0% *p*)
Cart-mounted MMS	4.9	−0.1	0.1	Yes
Backpack LiDAR	5.2	+0.2	0.2	Yes
SLAM-based backpack	5.2	+0.2	0.2	Yes
Handheld LiDAR	5.3	+0.3	0.3	Yes
SLAM-based handheld	5.1	+0.1	0.1	Yes
Terrestrial LiDAR	5.0	–	–	–

**Table 12:** Summary of barrier-related performance.

Platform	Meets Positional-Accuracy Criterion	Detects 2 cm Steps	Represents Longitudinal Grade (±1%)	Detailed Barrier Geometry
Terrestrial LiDAR	○	○	○	○
Cart-mounted MMS	○	○	○	△
Backpack LiDAR	○	X	○	○
SLAM-based backpack	○	○	○	○
Handheld LiDAR	○	X	○	△
SLAM-based handheld	○	○	○	○

(○: satisfies criterion; △: partially satisfies/conditional; X: not suitable).

**Table 13:** Recommended platform deployment and combinations by environment.

Environment/Purpose	Primary Platform	Supplementary Platform	Expected Benefits
Open-sky, long baseline reference tracks	Cart-mounted MMS	Backpack	Continuous trajectories; high vertical precision
Facility-dense neighborhood streets	Backpack	Handheld	Enhanced planimetric alignment and mapping continuity
Hard-to-access areas and around street furniture	Handheld	Backpack	Reinforcement of fine features; improved boundary depiction
Road–sidewalk boundary connections	Cart-mounted MMS (reference) + backpack/handheld in combination	-	Ensured boundary continuity and relative alignment

## Data Availability

The data presented in this study are available from the corresponding author upon reasonable request.
